# Mathematical modelling of contact dermatitis from nickel and chromium

**DOI:** 10.1007/s00285-019-01371-2

**Published:** 2019-06-13

**Authors:** J. P. Ward, S. J. Franks, M. J. Tindall, J. R. King, A. Curtis, G. S. Evans

**Affiliations:** 1 Department of Mathematical Sciences, Loughborough University, Loughborough, LE11 3TU UK; 2 School of Mathematical Sciences, University of Nottingham, Nottingham, NG7 2RD UK; 3 Department of Mathematics and Statistics, University of Reading, Reading, Berkshire RG6 6AX UK; 4 Institute for Cardiovascular and Metabolic Research, University of Reading, Reading, RG6 6AA UK; 5 Health and Safety Laboratory, Harpur Hill, Buxton, Derbyshire SK17 9JN UK

**Keywords:** Contact dermatitis, Metal ions, Immune response, Mathematical model, Numerical solution, 92C50

## Abstract

Dermal exposure to metal allergens can lead to irritant and allergic contact dermatitis (ACD). In this paper we present a mathematical model of the absorption of metal ions, hexavalent chromium and nickel, into the viable epidermis and compare the localised irritant and T-lymphocyte (T-cell) mediated immune responses. The model accounts for the spatial-temporal variation of skin health, extra and intracellular allergen concentrations, innate immune cells, T-cells, cytokine signalling and lymph node activity up to about 6 days after contact with these metals; repair processes associated with withdrawal of exposure to both metals is not considered in the current model, being assumed secondary during the initial phases of exposure. Simulations of the resulting system of PDEs are studied in one-dimension, i.e. across skin depth, and three-dimensional scenarios with the aim of comparing the responses to the two ions in the cases of first contact (no T-cells initially present) and second contact (T-cells initially present). The results show that on continuous contact, chromium ions elicit stronger skin inflammation, but for nickel, subsequent re-exposure stimulates stronger responses due to an accumulation of cytotoxic T-cell mediated responses which characterise ACD. Furthermore, the surface area of contact to these metals has little effect on the speed of response, whilst sensitivity is predicted to increase with the thickness of skin. The modelling approach is generic and should be applicable to describe contact dermatitis from a wide range of allergens.

## Introduction

Contact dermatitis is a common condition in the workplace. Eurostat data estimated the incidence of contact dermatitis as 5.5 cases per 1000 employees (De Craeker 2008). In the UK, annual incidence rate of 12.9 per 100,000 workers has been reported (Cherry et al. 2000). It commonly arises in occupations using soaps and cleaners, wet work, rubber chemicals and the use of Personal Protective Equipment (PPE) (in particular, disposable gloves which have an occlusive effect on the skin when worn for long periods) (HSE 2017). Exposure of the skin to metal allergens, via metal ion carriers (MIC) occurs in the construction, jewellery and paint manufacturing industries with nickel (occurring in its most active state nickel ($\hbox {II}^{+}$)) being the most common causative agent. Other metals such as Hexavalent Chromium (CrVI) are also regarded as a risk for dermatitis although recent statistics suggest a decreased incidence of dermatitis associated with electroplating work and the reduced use of chromates in cement (HSE 2017). EPIDERM data during the period 1996–2017, shows that approximately 37% of cases of contact dermatitis were induced by an allergic response and 44% by an irritant one (the remainder was mixed or unspecified; HSE 2017).

Hexavalent chromium (CrVI) exposure results in an immediate localised irritant response to the exposed skin, caused by the reduction of Cr(VI) to Cr(III), whilst nickel causes an allergic immune response. Both metals result in a localised inflammation of the healthy skin tissue. Nickel sulphate is known to cause allergic contact dermatitis (ACD) by directly interacting with specific histidine residues in the human Toll-like receptor 4 (TLR4), which normally act as an innate immune receptor for bacterial lipopolysaccharide (LPS). In doing so it mimics pathogen-associated molecular patterns (PAMPs) activating intracellular pro-inflammatory signalling pathways. There is other evidence that nickel may bind to the major histocompatibility molecules (MHC) and as a consequence, cross linking surface receptors in T-cell receptors activate their cytotoxic responses. The development of ACD, a delayed hypersensitivity response therefore appears to require activation of the innate immune system (Schmidt and Goebeler 2015), i.e. the nonspecific defence mechanisms that are activated immediately or within hours of an antigen’s appearance in the body. It has been estimated that approximately 10–15% of the human population suffers from contact hypersensitivity to metals (Budinger and Hertl 2000), but this allergic response is considerably more common in women (10%) than men (2%) (Peltonen 1979). The adsorption and excretion of metals is controlled by genetic factors in humans and single changes in DNA nucleotide sequence of specific genes can affect uptake metabolic pathways for handling metals, accounting for individual variability to metal toxicity (Ng et al. 2015).

Exposure in the workplace is usually repetitive and hence, difficult to prevent (Kanerva et al. 2000). Whilst treatment for the condition can be as simple as removing the source, in many occupations this is not always possible and hence further understanding of the biological mechanisms, both local to the contact area and associated with the immune system, is required in order to provide better treatment protocols. In the mathematical modelling to follow we aim to get a better understanding of how nickel and chromium elicits contrasting immunological responses and, for example, how skin structure and area of exposure effects the outcome.

Human skin consists of four main regions: the stratum corneum, the viable epidermis, the dermis and the subcutaneous layer. The stratum corneum is the outermost layer and plays a key role as a barrier to the penetration of molecules. Keratinocytes occupy approximately 95% of the epidermal layer and are produced in the stratum basale, the region dividing the epidermal and dermal regions. The dermis consists of a matrix of connective tissue housing the vasculature, lymphatic system, hair follicles and sweat glands. The subcutaneous layer consists of fatty (adipose) tissue; although this layer consists of living cells, there seems to be little evidence to suggest that it experiences a significant inflammatory response during metal ion exposure, unlike the epidermis and dermis.

Whilst both the epidermal and dermal layers are exposed to the effects of the irritant metal, different host responses result from exposure to chromium and nickel. Chromium exposure leads to a localised irritant response where the immune response is relatively rapid (within minutes) and involves the action of a host of immune cells, including macrophages and neutrophils, and numerous cytokines produced both by affected keratinocytes and by immune cells (Williams and Kupper 1996). The cytokines perform a number of functions, acting as promoters and inhibitors of immunological activity and as chemoattractants recruiting more immune cells into the compromised area. For the work to be undertaken here we are primarily interested in the role of the pro-inflammatory cytokine, interleukin-1$\alpha$ (IL-1$\alpha$) (released from damaged keratinocytes), based on experimental measurements of its activity in response to both chromium and nickel exposure (Curtis et al. 2007; Franks et al. 2008). The immune cells employ a range of cytotoxic processes to clear damaged keratinocytes in the irritated zones, both specific (applied directly to affected cells) and non-specific (killing all cells in the vicinity).

Nickel causes a similar localised response to chromium VI, but in addition, initiates a T-lymphocyte mediated delayed type-IV hypersensitivity reaction which occurs after previous exposure and sensitisation to the metal. High molecular-weight protein antigens (e.g. from an invading pathogen) typically stimulate humoral (i.e. that which is mediated by macromolecules found in the extracellular fluid) and innate immunity (i.e. the nonspecific defence mechanisms that are activated immediately or within hours of an antigen’s appearance in the body) when specialised antigen presenting cells such as Langerhans cells encounter these foreign antigens in the skin. They then migrate to local lymph nodes presenting the antigen to other immune cells e.g. T-cells, activating cytotoxic responses against pathogens. However, nickel, by activating these cytotoxic responses may stimulate ACD independently of foreign antigens. For approximately 2 days, the activated T-cells remain in the lymph node undergoing two to three cell divisions. The cells then disperse into the blood stream and reach the affected area of skin.

Following removal of these two metals, the skin may undergo a process of regrowth and repair, the latter mediated by macrophages and fibroblasts. Furthermore, activated T-cells remain in the body (at a low level), and will rapidly reactivate and divide on subsequent exposures to the MIC. We note that the recovery processes will not be taken into account in the modelling below.

There is an extensive literature regarding the transport of agents through skin (see Jepps et al. 2013, Naegel et al. 2013 and references therein), skin disease (see Mollee and Bracken 2007, Tanaka and Ono 2013 and references therein), skin disease (Mollee and Bracken 2007; Tanaka and Ono 2013; Zhang et al. 2015; the latter specifically focusing on dermatitis) and immunological response on a molecular level (Dominguez-Hüttinger et al. 2013; Le et al. 2015; Lorenzi et al. 2015; Palsson et al. 2013). To date there has been limited mathematical modelling of contact dermatitis in the literature. The most closely related to that of the work presented here is that of Dominguez-Hüttinger et al. (2017) who developed a dynamic multiscale model of atopic dermatitis to understand the interaction between known genetic and environmental factors affecting the disease. Using a system of three nonlinear ODES to describe the interactions between the skin barrier, immune response and the effect of environmental factors, the authors demonstrate their model exhibits bistable behaviour in predicting either eradiction or further development of the condition. In other work, Döpfer et al. (2012) used an ODE infectious disease model to demonstrate the importance of detecting and treating dermatitis lesions quickly in reducing the spread of digital dermatitis in dairy cattle. Maxwell et al. (2014) studied mathematical models of the CD8+ T-cell response in the presence of a sensitising chemical, a key pathway in the contact dermatitis response; they discuss the importance of using such mathematical models to replace animal models in future work.

Our work is the first we are aware of which takes a spatiotemporal approach to understanding the key features of acute and chronic contact dermatitis, namely sensitisation and the immune response on the whole body as well as local skin level. In Sect. 2 we present the mathematical model which provides a description of the localised skin response in the dermal and epidermal regions to insult by a toxic agent, either chromium or nickel, coupled with the effects of a time-mediated immune-system response, over the first few days of contact with the MIC. The spatio-temporal model developed allows the localised skin reaction to be compared between irritant and allergic cases and the effects of a T-lymphocyte immune-system response to be elucidated. A simplified form of the model (excluding spatial and immune response effects) has been shown to be in good agreement with in vitro experimental data (Curtis et al. 2007; Franks et al. 2008) regarding the exposure of keratinocytes to nickel and chromium. Results from that work are used to parameterise our model, allowing us to test the effect of each mechanism, local and non-local, on the localised skin response to metal exposure.

## Model description

### Model formulation

**Fig. 1:**
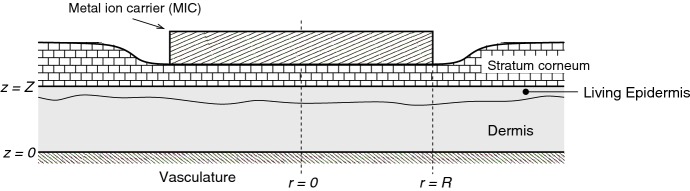
Schematic of the irritated skin region (proportions not to scale). The metal ion diffuses through the stratum corneum (which may be partly eroded) into the epidermis and dermis (indicated in grey), the latter region contains the vasculature which acts as a source for newly recruited immune cells and as a sink for cytokines and metal ions. The model domain is the grey region

**Fig. 2:**
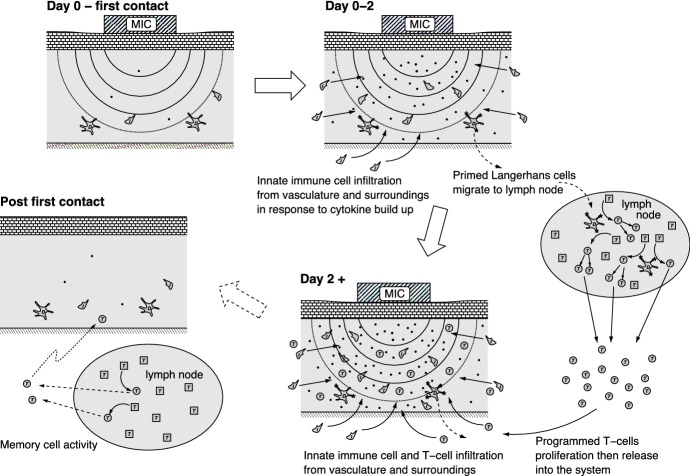
Schematic of the irritant and allergenic process as described by the model for the first contact case. The figures show the skin region in the vicinity of the metal ion carrier (MIC), with contours depicting metal ion gradients. On initial contact there is a background level of innate cells, Langerhans cells and cytokines (shown as “bullet”s). Over the first 2 days, innate immune cells respond to chemokine signals and migrate into the infected skin region, whilst primed Langerhans cells migrate to the lymph nodes carrying ion asssociated antigens (shown as “star”s). Within the lymph nodes, the antigen is presented to naive T-cells, activating them to be programmed T-cells. These proliferate and are then released into the system from around Day 2 eventually migrating to the exposed skin region. On removal of the MIC, the skin returns to the pretreated state with a low level of activated immune cell activity. This represents the initial conditions for the second contract case, for which the ‘Day 2+’ scenario is expected to arise notably sooner. Note skin cell death is not shown in the figure

The model proposed will track the evolution of skin damage in response to ion toxicity, innate immune cell (IC) activity, T-cell activity and response regulation by a generic cytokine/chemokine. For simplicity, we will henceforth refer to innate immune cell activity as the innate response (density *I*), T-cell activity as the adaptive (humoral) response (density *T*) and the cytokine/chemokine as just cytokines (density *c*). The focus will be on the initial responses to the MIC, i.e. up to about 6–8 days; as a first approximation we will not take into account the healing processes that will be occurring, on the assumption that these processes are secondary in the initial phases of the allergic response. The model will be developed for a general spatial geometry, based on the setup depicted in Fig. 1. The ions are sourced from the MIC on the skin surface and diffuse through the dead cell layers of the stratum corneum into the living regions that are of modelling interest, namely the dermis and epidermis (the shaded grey region in Fig. 1). Although the dermis and epidermis are distinct zones, we will assume for simplicity that the modelling domain is homogeneous and that it is bounded between $z=0$ (the location of the vasculature) and $z=Z$ (the live skin and stratum corneum interface). We will ignore the possible contributory factor of MIC abrasion on the inflammation process. The model will be studied in Sect. 3 mainly in 1-D (depth based) and in 3-D with radially symmetry assumed ($r\in [0,\infty ])$. The sequence of events, for which the model is intended to describe, is illustrated in Fig. 2. The scenario depicted is for the first-contact case where there are no activated T-cells present initially. Contact with the ion leads to a cascade of immune activity, firstly innate and then adaptive, whereby eventual removal of the ion carrier leads to a situation of low level adaptive immune activity in readiness for second contact. There will be a number of parameters in the model and in the interest of being systematic with their naming we define $\beta _{{\mathrm{ab}}}$ and $\delta _{{\mathrm{ab}}}$ to be the rate constants for the birth and death, respectively, of “*a*” due to “*b*”; $\delta _{{\mathrm{a}}}$ is the natural decay rate constant of species *a*. Details on dimensional parameter values sourced and estimated from the literature are given in Table 2. Table 1 lists the dependent variables in the model.

It is assumed that the ‘skin’ is made up of living cells such as keratinocytes (volume fraction *n*), breakdown product of dead cells (*p*) and extracellular fluid (fixed volume fraction $w_0$); the immune cells, although present, are assumed to occupy a very small fraction of space. We note that *p* is a product of cytolysis (i.e. the dissolution of cells, especially by an external agent), whether by apoptosis or necrosis, which will be treated as distinct from the live cell and extracellular fluid. Hence, the volume fractions satisfy

1 $$\begin{aligned} n\,+\,p~=~1-w_0. \end{aligned}$$

The volume fraction of live cell fraction evolves according to

2 $$\begin{aligned} \frac{\partial n}{\partial t} = -K_d\,n, \end{aligned}$$

where

3 $$\begin{aligned} K_d ~=~ (\delta _{{\mathrm{ni}}}+\delta _{{\mathrm{nTi}}}H(F(A_e,c)-1) T)A_n \,+\, \delta _{{\mathrm{ng}}}g. \end{aligned}$$

This accounts for death due to the toxic effects of the intracellular ion ($\delta _{{\mathrm{ni}}}A_{i}$), direct killing of ion containing cells ($\delta _{{\mathrm{nTi}}}H(F(A_e,c)-1)T)A_n$) by activated T-cells and the cytotoxic products (henceforth referred to as granules) released by the neutrophil and other granulocytes component of the innate immune system ($\delta _{{\mathrm{ng}}}g$, with *g* being the concentration of granules). The function *H*(*x*) is the Heaviside function, such that $H(x) = 1$ for $x \ge 0$ and $H(x)=0$ for $x < 0$; this function is a simple representation of the nonlinear activation response by immune cells to a range of cytokines (Callard et al. 1999). This term appears in several equations, ensuring that the model has a steady-state representing a healthy state and that there is no auto-immune activity in the absence of the irritant. T-cell activation is assumed to occur when $F(A_e,c) > 1$, where

4 $$\begin{aligned} F(A_e,c) ~=~ \left( \frac{A_e}{A_{e_i}}\right) ^{m_T} \,+\, \left( \frac{c}{c_{T}}\right) ^{m_c}, \end{aligned}$$

which is a measure of the combined stimulus on T-cells from the metal ion *i* and cytokines. On death, it is assumed that the entire volume of live cells become dead cell debris so it follows that

5 $$\begin{aligned} \frac{\partial p}{\partial t}= & {} K_d \,n, \end{aligned}$$

from Eqs. (1) and (2).

**Table 1:** List of model variables and their interpretation

Variable	Description	Variable	Description
*n*	Live cell volume fraction	*p*	Dead cell volume fraction
$A_e$	Extracellular ion conc.	$A_N$	Live cell ion conc.
$A_p$	Dead cell ion conc.	*c*	Cytokine concentration
*I*	Innate immune cell density	*g*	Granule (cytotoxic product) concentration
*T*	Programmed T-cell density	*L*	Lymph node activity

Ions can exist in three states: (1) an extracellular, diffusible state (concentration $A_e$); (2) bound to living cells (concentration $A_n$), inducing the cellular response; and (3) bound to cell debris (concentration $A_p$); chromium (Shrivastava et al. 2002) and nickel (Hausinger 1993) ions have been shown to readily traverse the cell membrane. The ions bound to the cell debris that are consumed by the macrophages are no longer available to be taken up by cells. The extracellular ion is removed by dendritic cells with rate constant $\delta _{eD}$. In our model we assume that the ions are a component of the antigen as it activates the same responses independently of protein allergens. We also assume that there is an ion exchange (both passive and active) between the extra- and two intracellular phases and that there is partitioning between these states (with partition coefficients $\mu _{n}$ and $\mu _{\rho }$). Such partitioning occurs in many cellular-chemical systems as discussed by Franks et al. (2008). The governing equations for the extra- and intracellular metal ions can therefore be written as

6 $$\begin{aligned} \frac{\partial (nA_n)}{\partial t}= & {} -k_n n(A_n - \mu _nA_e) \,-\, K_d nA_n, \end{aligned}$$

7 $$\begin{aligned} \frac{\partial (pA_p)}{\partial t}= & {} -k_p p (A_p - \mu _p A_e) \,+\, K_d nA_n, \end{aligned}$$

8 $$\begin{aligned} w_0 \frac{\partial A_e}{\partial t}= & {} D_e w_0\,\nabla ^2 A_e \,+\, k_n n(A_n - \mu _n A_e) \,+\, k_p p(A_p - \mu _p A_e) \,-\, \delta _{{\mathrm{eD}}} A_e, \nonumber \\ \end{aligned}$$

where $k_{n}$ and $k_{p}$ are the ion exchange rates and $D_e$ is the respective diffusion coefficient of extracellular ions; we note these parameters are different for nickel and chromium. We further note that we do not distinguish between Cr(VI) and Cr(III), whereby the reduction to Cr(III) produces the free radicals that damages cells; this is reasonable provided that Cr(III) forms only a small part of the chromium ion reservoir.

Live cells (e.g. keratinocytes) with metal bound to them are under stress. They respond to this stress by releasing a variety of chemokines and cytokines that have a range of functions: chemoattractant, activator of immune cells, growth promoters and immunosupressors. For simplicity we consider a single pro-inflammatory-type generic cytokine, with diffusion rate $D_{c}$. Cells release cytokines at a low-level background rate ($\beta _{cn}$), but the production rate is enhanced when the cell is stressed (due to bound ions, $\beta _{ci}nA_n$). Further production of cytokines is carried out by activated innate immune cells (activation induced when cytokines concentration is above a critical threshold, $c_I$) and by T-cells in response to the ion or associated antigen (a Monod kinetic form is assumed with a maximum production rate per T-cell of $\beta _{{\mathrm{cET}}}$). Taking all of these mechanisms into account, the equation governing the cytokine concentration is therefore given by

9 $$\begin{aligned} w_0\frac{\partial c}{\partial t}= & {} D_c w_0\, \nabla ^2 c \,+\, \beta _{{\mathrm{ci}}} n A_n \,+\, \beta _{{\mathrm{cn}}} n \,-\, \delta _{{\mathrm{c}}} w_0 c\nonumber \\&+ \, \beta _{{\mathrm{cET}}} w_0 \frac{A_e}{(A_{c_i}+A_e)} T \,+\, \beta _{{\mathrm{cI}}} w_0 H(c-c_I) I, \end{aligned}$$

where we have included a natural cytokine decay rate (constant $\delta _{c}$). This rate will of course vary between signalling molecules but can be viewed here as an average decay rate of similar sets of cytokines. In the absence of any metal ion and neglecting any contribution from T-cells, the spatially homogeneous steady state, $c_{ss}$, is

10 $$\begin{aligned} c_{ss} ~=~ \frac{(1-w_0)\beta _{{\mathrm{cn}}}}{w_0 \delta _{{\mathrm{c}}}}, \end{aligned}$$

giving an upper bound for the background level of cytokine. We assume the immune cell activation thresholds are such that $c_{ss}<c_I$ and $c_{ss} < c_T$.

Innate immune cells move through the skin structure by diffusion and chemotaxis, in response to the cytokine (e.g. Il-8$\alpha$) concentration gradients, at a rate described by the flux $\varvec{J_I}$. Degradation of extracellular matrix molecules such as fibronectin may also act to stimulate the movement and migration of these cells. There is also a loss of innate immune cells due to natural wastage ($\delta _{I} I$), “exhaustion” from production of granules (produced when $c > c_I$). We further assume that the natural death rate of activated immune cells is reduced (at rate $\delta _{{\mathrm{I}}}(1-\delta _{{\mathrm{Ic}}}$)). The equation for *I* is thus

11 $$\begin{aligned} w_0 \frac{\partial I}{\partial t}= & {} - \nabla \cdot \varvec{J_I} \,+\, w_0 \beta _{I} H(c-c_I)I\nonumber \\&\,-\, \left( \frac{(\beta _{{\mathrm{gc}}} H(c-c_I) +\beta _{{\mathrm{gi}}} nA_n)}{M_c} \,+\, \delta _{{\mathrm{I}}}(1-\delta _{{\mathrm{Ic}}}H(c-c_I))\right) w_0 I. \qquad \end{aligned}$$

The process of immune cell movement through the skin structure employs a range of integrins to gain purchase on the various components of the extracellular matrix (ECM) (Luster et al. 2005). We assume that ECM is present where there is live skin and adopt an immune cell flux term that is proportional to the live cell fraction *n*. Furthermore, we assume that only activated immune cells respond to chemotactic cues. The flux $\varvec{J_I}$ is therefore given by

$$\begin{aligned} \varvec{J_I} ~=~ - w_0\, n\, D_I \nabla I \,+\, w_0\,n \,\chi _IH(c-c_I) I \nabla c, \end{aligned}$$

where the diffusion $D_I$ and chemotactic $\chi _I$ rate coefficients will be assumed constant for simplicity, though, in general, they will be functions of the chemokine concentrations (see Lin et al. 2004). The renewal of innate immune cells is via transport from the vasculature, satisfying the flux condition (17) at $z=0$.

The innate immune cells (specifically, the proportion that are neutrophils and granulocytes) when activated (i.e. when $c>c_I$) release cytotoxic granules and free radicals when in direct contact with ion-bound stressed cells. These granules get consumed during the keratinocyte cell-killing process and through natural decay, hence

12 $$\begin{aligned} w_0 \frac{\partial g}{\partial t}= & {} w_0\left( (\beta _{{\mathrm{gc}}} H(c-c_I) +\beta _{{\mathrm{gi}}} nA_n)I \,-\,\frac{\delta _{{\mathrm{ng}}}}{M_g} gn-\delta _{{\mathrm{g}}} g \right) . \end{aligned}$$

We note in the absence of ions and activated innate immune cells $g=0$ is a stable steady-state.

The T-cell response is two-fold, involving, firstly, priming and an initial proliferation phase in the lymph nodes (a response that takes about 2 days following ion exposure) and, secondly, proliferation at the site of contact (in response to extracellular ion concentration and cytokines when they are above the thresholds $A_{e_i}$ and $c_T$, respectively, in Eq. (4)). T-cells enter the system at $z=0$ at a flux proportional to the dimensionless “lymph-node output activity” *L* (boundary condition (18)). As with innate immune cells, the T-cell flux $\varvec{J_T}$ is governed by random motion and, when activated, chemotaxis up cytokine gradients, hence

13 $$\begin{aligned} w_0\frac{\partial T}{\partial t}= & {} -\nabla \cdot \varvec{J_T}\, +\, w_0 ( \beta _{{\mathrm{T}}} H(F(A_e,c)-1) \,-\, \delta _{{\mathrm{T}}}) T, \end{aligned}$$

and

$$\begin{aligned} \varvec{J_T} ~=~ - w_0\, n\, D_T \nabla T \,+\, w_0\,n \,\chi _{Ti} H(F(A_e,c)-1) \,T \,\nabla c, \end{aligned}$$

again assuming that ECM around the living cells are required for cell motion. The dimensionless variable *L*(*t*) governs inward flux of T-cells into the skin region. Prior to ion contact $L=0$ so that $T=0$. The value of *L*(*t*) will become non-zero following ion contact and will reach a peak when the lymph nodes are most actively releasing newly programmed T-cells. Following removal of the irritant, the value of *L*(*t*) will drop to a basal level $\varepsilon$, so that there will be a continued presence of programmed T-cells in the skin, albeit at a much lower level. The activity in the lymph nodes are assumed to be governed by a Michaelis–Menten function of the total amount of extracellular ions $A_e$ (assumed to be proportional to the amount picked up by dendritic cells) over the previous few days. The passage of dendritic cells to the lymph nodes and the recruitment of naive T-cells typically takes around 2 days (Stoitzner et al. 2003), though, we will assume the timescale for this process can be distributional, given by function $k(\tau )$. To describe the winding down process of $L\rightarrow \varepsilon$, whilst permitting $L=0$ to be a steady-state as well, we use, for simplicity, a logistic term. The evolution of *L*(*t*) is assumed to follow

14 $$\begin{aligned} \frac{dL}{dt}= & {} \lambda \left( \frac{\displaystyle {w_0\int _V \int _0^{\infty } k(\tau ) \,A_e(\varvec{x},t-\tau ) \,\text{ d }\tau \, \text{ d }V}}{\displaystyle {K_{L_i}+w_0\int _V \int _0^{\infty } k(\tau )\, A_e(\varvec{x},t-\tau ) \, \text{ d }\tau \,\text{ d }V}} \,+\, L\,(\varepsilon -L) \right) , \end{aligned}$$

where $K_{L_i}$ is approximately the number of ions (in mols) at which activity of the lymph node is half its maximum and *V* is the skin domain. In the biologically relevant case of $\varepsilon \ll 1$, the maximum value of *L*(*t*) will be a little over unity. The kernel $k(\tau )$ in the integrals of (14) is assumed to have the following property

$$\begin{aligned} \int _0^\infty k(\tau ) \,\text{ d }\tau ~=~ 1, \end{aligned}$$

and that the mean time ${\bar{\tau }}$ for programmed T-cell release following ion uptake by dendritic cells is

15 $$\begin{aligned} {\bar{\tau }} ~=~ \int _0^\infty \tau \,k(\tau ) \,\text{ d }\tau . \end{aligned}$$

We will mainly focus on the simplest case in which $k(\tau ) = \delta (\tau -{\bar{\tau }})$, where $\delta (.)$ is the Dirac delta function, i.e. this process takes ${\bar{\tau }} \approx 2$ days.

### Boundary and initial conditions

To simulate the in vivo scenario it is assumed that a metal ion carrier is applied at $t=0$. At this time the skin is perfectly healthy and the concentration of macrophages, T-cells and cytokines are at a “background” steady state. The model will be studied in one-dimension ($0<z<Z$) and in three-dimensions assuming radial symmetry ($0<z<Z$ and $0 \le r < \infty$). In the latter case $r=0$ is the coordinate of the MIC’s centre as illustrated in Fig. 1. The boundary conditions are

16 $$\begin{aligned} \text{ at } z=0&A_e=0,~-\!w_0 D_c \frac{\partial c}{\partial z}=- w_0 Q_c c, \end{aligned}$$

17 $$\begin{aligned}&\varvec{J_I}\cdot \varvec{\Gamma _z} = w_0 \,n \,Q_I, \end{aligned}$$

18 $$\begin{aligned}&\varvec{J_T}\cdot \varvec{\Gamma _z} = w_0\, n \,Q_T \,L(t), \end{aligned}$$

19 $$\begin{aligned} \text{ at } z=Z&~~~&\frac{\partial c}{\partial z} = \varvec{J_I}\cdot \varvec{\Gamma _z}=\varvec{J_T}\cdot \varvec{\Gamma _z}=0,~ \left\{ \begin{array}{ll} A_e=A_{surface} ~~&{} 0 \le r \le R\\ \frac{\partial A_e}{\partial z}=0 &{} r> R \end{array} \right. \end{aligned}$$

20 $$\begin{aligned} \text{ at } r=0&\frac{\partial A_e}{\partial r}=\frac{\partial c}{\partial r} = \varvec{J_I}\cdot \varvec{\Gamma _r}=\varvec{J_T}\cdot \varvec{\Gamma _r}=0, \end{aligned}$$

21 $$\begin{aligned} \text{ as } r \rightarrow \infty&A_e\rightarrow 0,~c\rightarrow c_I(z), ~I\rightarrow I_I(z),~T\rightarrow T_\infty (z,t), \end{aligned}$$

where $\varvec{\Gamma _i}$ for $i=\{r,z\}$ is the unit vector in direction *i* and the $Q_*$s are mass transfer coefficients. Here, the influx rate of T-cells from the blood is assumed proportional to lymph node activity *L* and $r \in [0, R]$ is the region of metal ion infiltration at $z=Z$; due to the large aspect ratio of likely MIC diameter and skin thickness, *R* will be approximately the width of the MIC (see Fig. 1). At “large” $r > R$, we impose “far-field” distributions for cytokine $c_I(z)$, innate immune cells $I_I(z)$ and T-cells, $T_\infty (z,t)$, that reflect those for healthy tissue (being too far from the MIC source to be affected); these distributions thus satisfy

$$\begin{aligned} 0= & {} D_c w_0\,\frac{\partial ^{2} c_I}{\partial z^2} \,+\, \beta _{{\mathrm{cn}}} (1-w_0) \,-\, \delta _{{\mathrm{c}}} w_0 c_I, \\ 0= & {} D_I w_0 (1-w_0) \frac{\partial ^{2} I_I}{\partial z^2} \,-\, w_0 \delta _{{\mathrm{I}}} I_I, \\ w_0 \frac{\partial T_\infty }{\partial t}= & {} D_T^0 w_0 (1-w_0) \frac{\partial ^{2} T_\infty }{\partial z^2} \,-\, w_0 \delta _{{\mathrm{T}}} T_\infty , \end{aligned}$$

subject to the boundary conditions

$$\begin{aligned} \text{ at } z=0&\frac{\partial I_I}{\partial z}=-\frac{Q_I}{D_I},~~~ \frac{\partial T_\infty }{\partial z}=-\frac{Q_TL(t)}{D_T }, ~~~ D_c\frac{\partial c}{\partial z}=Q_c c,\\ \text{ at } z=Z&\frac{\partial I_I}{\partial z}=0, ~~~\frac{\partial T_\infty }{\partial z}=0, ~~~\frac{\partial c_I}{\partial z}=0. \end{aligned}$$

We assume that the ion starts infiltrating the completely healthy skin at $t=0$, hence

22 $$\begin{aligned}&\text{ at } \ \ t=0 \ \ \ n=1-w_0,~ I=I_I(z),~ T= T_I(z;L_0),~L=L_0,~ A_n=0, \nonumber \\&\quad A_e=0,~p=0,~p_g=0,~c=c_I(z),~g=0, ~A_p=0, \end{aligned}$$

where $I_I(z), T_I(z;L_0)$ and $c_I(z)$ are the steady-state distributions in the absence of the ion given by

23 $$\begin{aligned} I_I(z)= & {} Q_I \sqrt{\frac{1-w_0}{\delta _{{\mathrm{I}}} D_I}}\, \frac{ \cosh \left( \sqrt{\frac{\delta _{{\mathrm{I}}}}{D_I(1-w_0)}}(Z-z)\right) }{\sinh \left( \sqrt{\frac{\delta _{{\mathrm{I}}}}{D_I(1-w_0)}}\,Z \right) }, \end{aligned}$$

24 $$\begin{aligned} T_I(z; L_0)= & {} L_0 \,Q_T \sqrt{\frac{1-w_0}{\delta _{{\mathrm{T}}} D_T}}\, \frac{\cosh \left( \sqrt{\frac{\delta _{{\mathrm{T}}}}{D_T(1-w_0)}}(Z-z)\right) }{\sinh \left( \sqrt{\frac{\delta _{{\mathrm{T}}}}{D_T(1-w_0)}}Z \right) }, \end{aligned}$$

25 $$\begin{aligned} c_I(z)= & {} c_{ss} \left( 1 - \frac{Q_c \cosh \left( \sqrt{\frac{\delta _c}{D_c}}(Z-z)\right) }{Q_c \cosh \left( \sqrt{\frac{\delta _c}{D_c}}Z\right) + \sqrt{D_c \delta _c}\sinh \left( \sqrt{\frac{\delta _c}{D_c}}Z\right) }\right) , \end{aligned}$$

where $c_{ss}$ is given by (10) and

26 $$\begin{aligned} L_0 ~=~ \left\{ \begin{array}{ll} 0 ~~~&{} \text{ first } \text{ contact } \text{ with } \text{ allergen, }\\ \varepsilon ~~~&{} \text{ subsequent } \text{ contacts } \text{ with } \text{ allergen, } \end{array} \right. \end{aligned}$$

representing memory cell activity following previous contacts.

The full governing system of equations is summarised and non-dimensionalised in “Appendix A”. Non-dimensionalising was undertaken to aid in parameter estimation as well as numerical solutions of the system.

### Parameter values

We have parameterised our model with values from the literature where they have been available as shown in Tables 2 and 3. Where values have not been available, we have made informed estimates to the non-dimensional parameters by comparing the magnitude and importance of each of the relevant mechanisms in the model. The non-dimensional parameter values are shown in Table 4.

As is indicated from the gaps in Tables  2 and 3 there is considerable uncertainty in the dimensionless values used in the simulations. Inspection of these non-dimensional values shows that they vary across several order of magnitudes, whereby for the set shown we could eliminate certain terms that have minor effect and hence concentrate on a reduced system. There is some value in doing this and perhaps will be the subject of a future study; such a reduced system will consist of nonlinear PDEs and, in all likelihood, we will still be limited to numerical approaches for their study. Moreover, the simulations to follow reveal the emergence of boundary layers, providing additional challenges in the potential use of analytical methods. We have not considered such model reductions for the current study and have kept all the proposed terms in the equations, as there importance, or lack thereof, may become apparent with more data and experimentation.

**Table 2:** List of non-ion specific model parameters

Parameter	Description	Value	Units	Sources
*Z*	Skin thickness	0.2	cm	Laurent et al. (2007)
$w_0$	Extracellular water volume fraction	0.05	Dimensionless	Moore (1987)
${\bar{\tau }}$	Mean lymph node response time	2.0	Day	Mempel et al. (2004)
$\beta _{cn}$	SC cytokine background prod. rate	$1.5 \times 10^{-3}$	$\upmu \hbox {M day}^{-1}$	
$\beta _{cI}$	Activated IIC cytokine prod. rate	–	$\upmu \hbox {M day}^{-1}$	
$\beta _{ceT}$	Activated PTC cytokine prod. rate	–	$\upmu \hbox {M day}^{-1}$	
$\beta _{gc}$	Cytokine mediated granule prod. rate	–	$\hbox {day}^{-1}$	
$\beta _{gi}$	Ion induced granule prod. rate	–	$\hbox {day}^{-1}$	
$\beta _{Ic}$	Activated IC growth rate	1.6	$\hbox {day}^{-1}$	Ganusov et al. (2005)
$\beta _{T}$	Activated TC growth rate	1.6	$\hbox {day}^{-1}$	$=\beta _{Ic}$
$\delta _{c}$	Cytokine decay rate constant	3.2	$\hbox {day}^{-1}$	Franks et al. (2008)
$\delta _{ng}$	Granule induced SC death rate	–	$\upmu \hbox {M}^{-1}\hbox { day}^{-1}$	
$\delta _{nTi}$	PTC induced SC death rate	–	$\hbox {day}^{-1}\,\upmu \hbox {M}^{-1}$	
$\delta _{eD}$	Background ion removal rate const.	–	$\hbox {day}^{-1}$	
$\delta _I$	IIC death rate const.	1.2	$\hbox {day}^{-1}$	Brach et al. (1992)
$\delta _T$	PTC death rate const.	–	$\hbox {day}^{-1}$	
$\delta _g$	Granule decay rate const.	8.3	$\hbox {day}^{-1}$	Enrique et al. (1999)
$\lambda$	Lymph node activation rate const.	–	$\hbox {Day}^{-1}$	
$\varepsilon$	Long term lymph node activity	–	Dimensionless	
$c_I$	Cytokine conc. for IIC activation	–	$\upmu \hbox {M}$	
$c_T$	Cytokine conc. for PTC activation	–	$\upmu \hbox {M}$	
$M_g$	SC death per granule	–	Dimensionless	
$M_c$	IIC removed per granule produced	–	Dimensionless	
$D_c$	Cytokine diffusion rate	0.10	$\hbox {cm}^2\hbox { day}^{-1}$	Nugent and Jain (1984)
$D_I$	IC diffusion coefficient	–	$\hbox {cm}^2\hbox { day}^{-1}$	
$\chi _I$	IIC chemotaxis coefficient	–	$\hbox {cm}^2\,\upmu \hbox {M}^{-2}\hbox { day}^{-1}$	
$D_T$	PTC diffusion coefficient	–	$\hbox {cm}^2\hbox { day}^{-1}$	
$\chi _{Ti}$	PTC chemotaxis coefficient	–	$\hbox {cm}^2\,\upmu \hbox {M}^{-2}\hbox { day}^{-1}$	
$Q_c$	Cytokine mass transfer const.	–	$\upmu \hbox {M cm day}^{-1}$	
$Q_I$	IIC mass transfer const. (m.t.c.)	–	$\upmu \hbox {M cm day}^{-1}$	
$Q_T$	PTC mass transfer const. (m.t.c.)	–	$\upmu \hbox {M cm day}{-1}$	

Known estimates are included with source

*IIC* innate immune cell, *PTC* programmed T-cell, *SC* skin cell

**Table 3:** Dimensional model parameter values that are different for nickel and chromium

Parameter	Description	*Cr*	*Ni*	Units	Sources
$\delta _{ni}$	Cell death rate from ion	$6.5 \times 10^{-4}$	$6.3 \times 10^{-5}$	$\upmu \hbox {M}^{-1}\hbox { day}^{-1}$	Franks et al. (2008)
$\beta _{ci}$	Live cell cytokine prod.	$7.8 \times 10^{-6}$	0	$\hbox {Day}{-1}$	Franks et al. (2008)
$k_n$	Live cell-ion binding rate	1.4	320	$\hbox {Day}^{-1}$	Franks et al. (2008)
$k_p$	Dead cell-ion binding rate	1.4	320	$\hbox {Day}^{-1}$	$k_p=k_n{}^\mathrm{a}$
$\mu _n$	Live cell ion partition coeff.	8.3	2.2	Dimensionless	Franks et al. (2008)
$\mu _p$	Dead cell ion partition coeff.	8.3	2.2	Dimensionless	$\mu _p=\mu _n{}^\mathrm{a}$
$\delta _{nTi}$	TC induced cell death rate	–	–	$\upmu \hbox {M}^{-2}\hbox { day}^{-1}$	
$\beta _{gi}$	Ion induced granule prod.	–	–	$\upmu \hbox {M}^{-1}\hbox { day}^{-1}$	
$K_{L_i}$	Lymph node activation const.	–	–	mol	
$A_{e_i}$	TC activation const.	–	–	mol	
$A_{c_i}$	TC cytokine prod. const.	–	–	mol	
$D_e$	Ion diffusion coefficient	1.3	2	$\hbox {cm}^2\hbox { day}^{-1}$	Pan et al. (2002) and Tantemsapya and Meegoda (2004)

$^\mathrm{a}$Assumed the values for dead and live cells are the same

**Table 4:** Dimensionless parameters and their values used in the “standard simulation”

Parameter	Value	Parameter	Value	Parameter	*Cr*	*Ni*
$A_{\text{ surface }}{}^{\mathrm{b}}$	0.3	$\beta _{cET}$	150	$\delta _{ni}\,^\mathrm{a}$	1.3	0.13
$\beta _{cI}$	0	$\beta _{gc}$	1	$\beta _{ci}\,^\mathrm{a}$	35	0
$\beta _{I}$	1.2	$\beta _{T}$	1.2	$k_n\,^\mathrm{a}$	2.8	640
$c_I$	1.5	$c_T$	$\infty ^{\mathrm{c}}$	$k_p\,^\mathrm{a}$	2.8	640
$\chi _{Iz}$	0.3	$\chi _{Ir}$	0.012	$\mu _n\,^\mathrm{a}$	8.3	2.2
$D_{cz}\,^\mathrm{a}$	5	$D_{cr}\,^\mathrm{a}$	0.2	$\mu _p\,^\mathrm{a}$	8.3	2.2
$D_{Iz}$	0.1	$D_{Ir}$	0.004	$D_{er}\,^\mathrm{a}$	2.6	4.0
$D_{Tz}$	0.1	$D_{Tr}$	0.004	$D_{ez}\,^\mathrm{a}$	65.0	100.0
$\delta _c$	6.4	$\delta _{eD}$	0.1	$\chi _{Ti z}$	0.1	0.1
$\delta _g\,^\mathrm{a}$	17	$\delta _I\,^\mathrm{a}$	2.4	$\chi _{Ti x}$	0.004	0.004
$\delta _{Ic}$	0.9	$\delta _{ng}$	10	$\delta _{nTi}$	2.5	2.5
$\delta _T$	0.006	$\varepsilon$	0.001	$A_{e_{i}}$	0.02	0.02
$\lambda$	1.4	$M_c$	100,000	$A_{c_i}$	0.1	0.1
$M_g$	0.001	$N_{remove}$	0.8	$\beta _{gi}$	300	300
$Q_c$	1	$Q_I$	$0.503^{\mathrm{d}}$	$K_{L_i}^{\mathrm{f}}$	1.6	1.6
$Q_T$	$6.186^{\mathrm{d}}$	$\hbox {SA}_{sim}$	3.141$^{\mathrm{e}}$			
$Z_D$	1					

$^\mathrm{a}$Values derived from those listed in Tables 2 and 3 assuming $A_0=1 ~\mu$M, ${\bar{\tau }}=2$ days, $Z = 0.2$ cm, $X=1$ cm and $w_0=0.05$; the rest are assumed values

$^{\mathrm{b}}$Equivalent to $A_e= 0.3\mu$M, which is in the intermediate zone of cell killing (Franks et al. 2008)

$^\mathrm{c}$Switches off T-cell sensitivity to cytokine, i.e. T-cell activation is governed only by ion presence

$^{\mathrm{d}}$Derived values that ensures $I=1$ and $T=1$ (when $L(0)>0$) on the $z=0$ boundary at $t=0$

$^{\mathrm{e}}$Contact surface area of $\pi$ in standard simulation, i.e. that of a circular object with unit radius

$^{\mathrm{f}}$The value is $5/\pi$, in 1-D simulations $K_{L_i}$ is changed according to $K_{L_i} \pi /\hbox {SA}_{sim}$, where $\hbox {SA}_{sim}$ is the assumed surface area

## Model analysis and results

Numerical solutions to the non-dimensional model (Appendix A) were undertaken using the method of lines, where central difference approximations were used for the spatial derivatives. The resulting system of ordinary differential equations approximated using the Numerical Algorithms Group (NAG) routine D02NJF, a routine that uses an implicit method designed for stiff problems that yields a sparse Jacobian matrix. For 1-D model simulations, the system was efficiently solved using a uniform grid, however, for the 3-D simulations (effectively 2-D by assuming radial symmetry), in order to deal with rapid variations in the *x* direction near the line $x = X$, a non-uniform grid was used with most of the points concentrated about this line. As will be demonstrated in Sects. 3.2 and 3.3, there are only small differences between the results from equivalent 1-D and 3-D simulations, so the majority of results discussed here are for the 1-D case.

There are a large number of parameters in this system with only a few that can be reliably estimated. The choice of parameters in Table 4 gave solutions that possess notable differences in responses between chromium and nickel and between first and subsequent contacts. To minimise any contrivances in our results, the only difference between the parameters regarding chromium and nickel are those measured by Franks et al. (2008) and the diffusion coefficients. In all simulations, the parameters used are those listed in Table 4, with any differences noted in the figure captions and main text. With the lack of detailed, relevant spatio-temporal data of events occurring in the skin during the first few days of contact, we cannot be certain how close the simulated results presented are to reality. The parameters are chosen so that the resulting simulated results show clearly a contrast in events between chromium and nickel ions and between first and second contacts, using as a basis the data measured by Franks et al. (2008). The model can produce results to describe the spatial-temporal evolution to a much higher level of detail than that which is currently achievable using experimental techniques and clinical observation. The aim of the simulations is to gain insights into this system with the aim of informing potential areas of experimental study.

### The standard simulation in 1-D

**Fig. 3:**
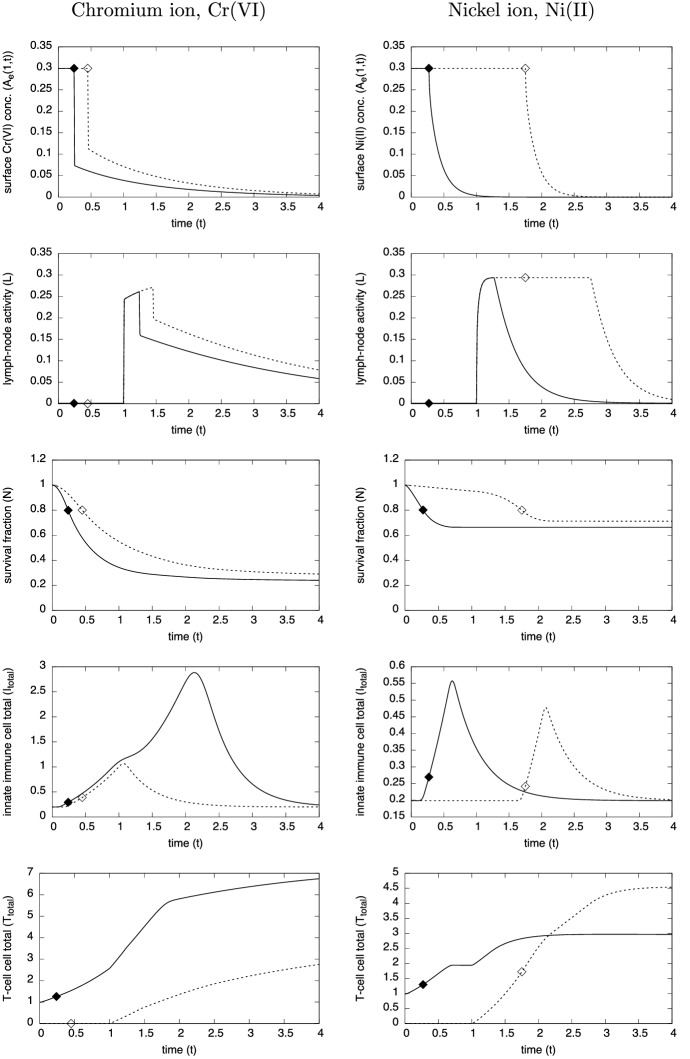
Plots of the evolution of surface ion concentrations (top), lymph node activity, survival fraction, total innate immune cell and T-cell densities (bottom) from initial contact of chromium (left) and nickel (right), through MIC removal (when $N=0.8$, indicated by the white diamond and black diamond) to $t=4$. First and second contacts are indicated by the dashed (with white diamond) and solid (with black diamond) curves, respectively. Parameters are as listed in Table 4

**Fig. 4:**
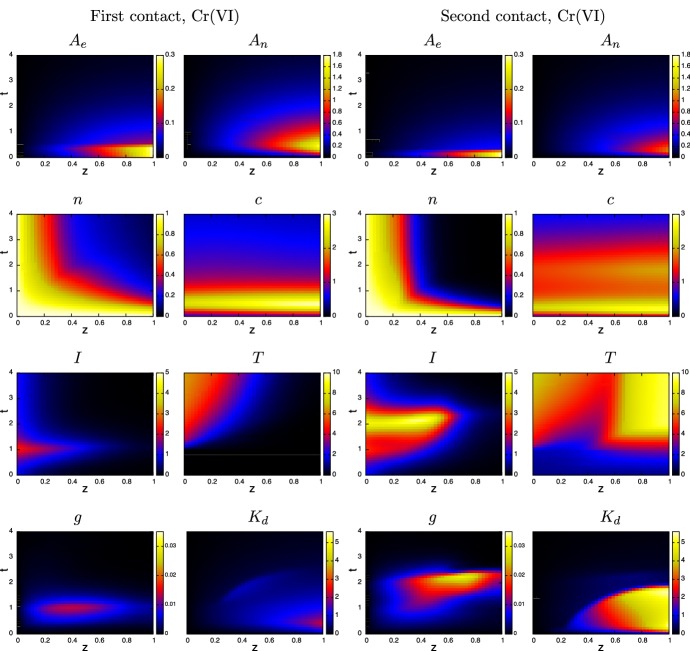
Evolution of the spatial distribution of the model variables over time $t=0$ to 4 (equivalent to 0–8 days) from contact with chromium (VI) in the 1-D case. The plots in the first two columns are for first-contact and those in the next two are second contact. The colour bars are the same for each of the corresponding variables. Parameters are as listed in Table 4 (color figure online)

**Fig. 5:**
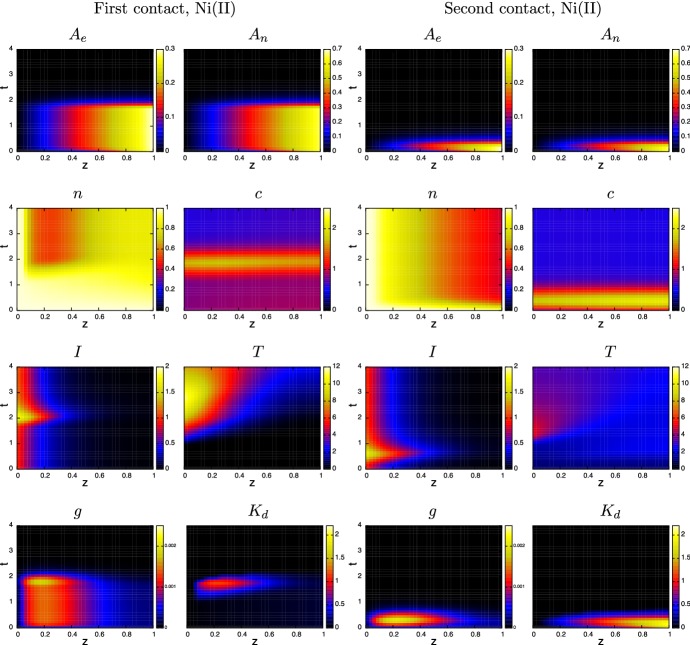
Evolution of the spatial distribution of the model variables over time $t=0$ to 4 (equivalent to 0–8 days) from contact with chromium (VI) in the 1-D case. The plots in the first two columns are for first-contact and those in the next two are second contact. The colour bars are the same for each of the corresponding variables. Parameters are as listed in Table 4 (color figure online)

The simulations presented here use the model parameters listed in Table 4 and referred to as the “standard simulation”. Figures 3, 4 and 5 show the evolution of the model variables over a non-dimensional period of $t \in [0,4]$ which is equivalent to a period of 8 days after contact with the metal ion. The MIC is in constant contact with the skin until the surviving fraction *N*(*r*, *t*) of cells, defined as

27 $$\begin{aligned} N(r,t) ~=~ \frac{1}{1-w_0}\int _0^1 n(r,z,t) \,\text{ d }z, \end{aligned}$$

falls below $N = N_{remove} = 0.8$. Although, the simulations here are in 1-D, the inclusion of the *r* variable is for the 3-D simulations discussed in Sect. 3.2. As expected for both metals, Fig. 3 shows that the period of time for MIC removal is longer in the first contact case, and considerably longer in the case of nickel. At the surface concentration of $A_e= 0.3$ (equivalent to 0.3 $\upmu$M), Cr(VI) is much more toxic than Ni(II), and in these simulations the toxicity of Cr(VI) is sufficient to cause MIC removal before adaptive immune response comes into play. As such, MIC removal was deemed to occur when 20$\%$ of the localised skin tissue became damaged. However, the pre-existence of specialist T-cells activates a rapid response and for both Cr(VI) and Ni(II) the MIC is removed by $t=0.3$ (about 15 h). For the first contact Ni(II) case, the concentration of ion is barely toxic and it is only when the adaptive response is activated and T-cells have successfully migrated from the blood stream to where Ni(II) concentration is highest does notable damage of the skin occur, consequently the MIC remains in contact until about $t = 1.75$ (equivalent to about 3.5 days).

The top two sets of plots in Fig. 3 highlight the consequences in the different properties of Cr(VI) and Ni(II) with regards to the rates of internal/external concentration equilibration and partitioning as measured by Franks et al. (2008); i.e. parameters $k_n, k_p, \mu _n$ and $\upmu _p$. The parameters indicate that Ni(II) equilibrates much faster than Cr(VI), yet the latter accumulates more in cells. This means that on removal of MIC, Cr(VI) levels will drop rapidly initially, but the longer time for the ion to leach out of cells means that its presence in the skin, though decaying, will remain for a few days. For Ni(II), the ion leaches out of cells faster, and eventually the ion is fully drained away from the skin within a day of removal. The longer presence of the Cr(VI) has the effect of prolonging immune and T- cell activity (bottom two sets of plots), which further kills cells for some time. Consequently, considerably more damage is predicted in the Cr(VI) case, with a surviving fraction of 20–30% as opposed to 60–70% in the Ni(II) case. The simulations suggest that in the Cr(VI) case the immune cell activity increases about 5 (first contact) to 15 (second contact) times that of the homeostatic state, which is significantly more than that in the Ni(II) case (approx 2–3 times). A further notable difference is the relative levels of T-cells present in skin between the metal ions on first and second contact. In the chromium case, T-cells accumulate more on second contact, whilst the opposite occurs for nickel. In the former case on first contact, the MIC has been removed and the ion depleted by the time T-cells infiltrate the contact region, hence the level of growth is much less on first contact. For nickel, lymph node activity is prolonged in the first contact case and hence more T-cells infiltrate the skin from the blood stream for a much longer period (compare the time-span at which *L* is at a maximum level in Fig. 3).

Figures 4 and 5 show “heat maps” of the simulated evolution of the spatial distribution of the main variables for chromium and nickel, respectively. The horizontal axis shows the depth variable *z* and vertical axis is time *t*. For each variable, the colour scale is the same for each metal ion so that the corresponding distributions can be compared. In each case, the ion distribution in the skin equilibrates fairly rapidly during initial contact and on removal of the MIC, characterised by the rapid drop in $A_e$ concentration. The equilibration of nickel ions between extracellular space and skin cells is much more rapid than chromium ions (see $A_n$ figures), with the mass transfer coefficient ratio being $k_n(\text{ Ni })/k_n(\text{ Cr }) \approx 230$; consequently nickel builds up and drains out quickly, whilst on removal of the MIC, chromium ions in cells acts an ion reservoir and hence they linger in the system. As can be seen from Fig. 4, immune cell activity in the chromium ion case is significantly enhanced on second contact (see plots of *I* and *T*), leading to greater damage of skin. On first contact, the relatively low immune cell activity suggest that skin cell death is largely due to toxicity of the chromium ions. On second contact, the rather delayed emergence of the innate immune cells (peaking around $t\approx 2$) suggests that death is due to ion toxicity and T-cell action; the notable peak in granules will have little effect as the skin cells have already died off there. Furthermore, T-cells are able to penetrate throughout the skin, unlike the innate cells, due to their initial presence in the skin and their relative longevity. In contrast, the immune cell activity in the nickel case becomes much more intense in the first contact than on second contact (see Fig. 5), particularly in T-cell activity. Here, nickel has less intrinsic toxicity than chromium, and skin cell death is associated with peaks in cytokines and hence caused by immune cell activity. However, on second contact, the area of skin cell death is beyond where innate immune cells have penetrated, so their role is secondary in this simulation.

We summarise the main results from this simulation. In the case of Cr(VI), the ions are sufficiently toxic to induce, after a short time ($t < 0.5$ or approximately 1 day), the removal of the MIC from the skin surface with only limited intervention from the immune system. However, the lingering of the chromium ions in the skin tissue leads to continued immune activity that can lead to further damage to the skin particularly near the viable skin surface. The data from Franks et al. (2008) suggest that nickel at equivalent concentrations is less toxic than Cr(VI) and induces negligible IL-1$\alpha$ production. Consequently, immune activity is dominated by T-cells, which, through there own cytokine production, activate immune cells to deal with the ion presence. On first contact, the absence of T-cells and relatively low cell death, means that the skin is tolerant to the ion presence for a relatively long time. On second contact, however, the T-cells react quickly leading to a fairly similar response to chromium exposure.

**Fig. 6:**
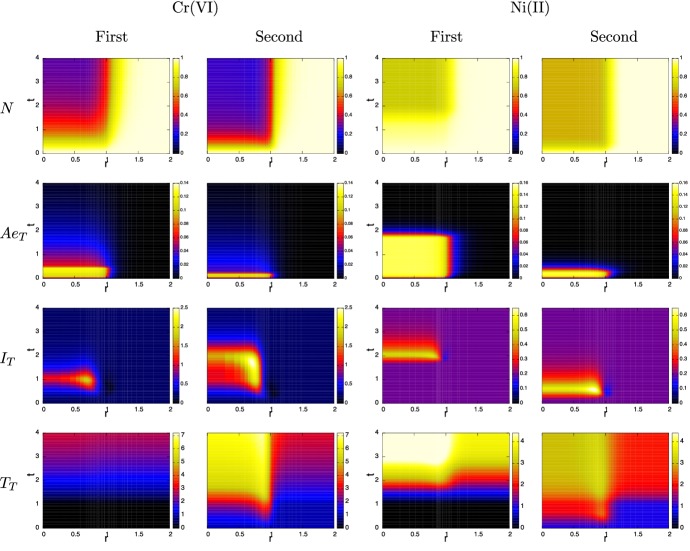
Evolution of the spatial distribution over *r* of $N, Ae_T, I_T$ and $T_T$ [from formulae (27) and (28)] over time $t=0$ to 4 (equivalent to 0–8 days) from contact with a disk of unit radius of chromium(VI) (left two columns) and nickel(II) in the standard case, simulated in 2D assuming radial symmetry. The colour bars are the same for each of the corresponding variables. Parameters are as listed in Table 4 (color figure online)

**Fig. 7:**
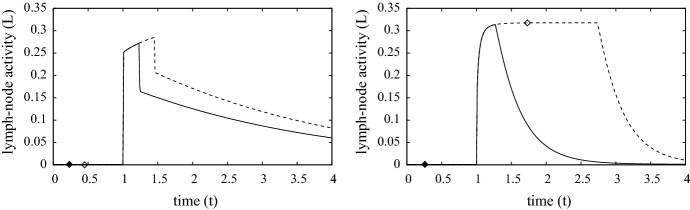
Plots of lymph node activity from contact with a MIC disk of unit radius containing chromium (left) and nickel (right), which was removed when when $\min _r\{N(r,t)\} =0.8$. First and second contacts are indicated by the dashed and solid curves, respectively, whereby the time points for MIC removal are indicated by white diamond (first exposure) and black diamond (second exposure). Parameters are as listed in Table 4

### The standard simulation in 3-D with radial symmetry

Figures 6 and 7 shows a number of results from the “standard simulation” in 3-D cylindrical geometry (though radial symmetry reduces this to a 2D system); this describes skin contact with a MIC circular disk of unit radius. All parameters are the same as those used in Sect. 3.1 including the contact surface area of $\pi$. The aspect ratio of skin thickness to contact region length is small (here about 0.1) and, in most circumstances, we would expect that the solutions of the 1-D model will agree well those of the 3-D model; this is indeed the case and is demonstrated further in Sect. 3.3. The main reason is that $A_e$ does not penetrate very far from the contact zone (no more than 2 mm), so that most of the cell death and immune activity is concentrated there with the periphery being largely unaffected. Figure 6 shows heat maps of the depth averaged radial distributions against time of the survival fraction (i.e. *N* as defined by (27)), external ion concentration $Ae_T$ defined as

28 $$\begin{aligned} Ae_{T}(r,t) ~=~ \int _0^1 A_e(r,z,t) \,dz, \end{aligned}$$

and the similarly defined averaged densities of innate immune cells ($I_T(r,t)$) and programmed T-cells ($T_T(r,t)$). The limited penetration and damage caused by the ions can be seen in the top two rows of graphs in Fig. 6. The cross-sectional profile along $r=0$ of $N, I_T$ and $T_T$ match very closely the corresponding curves in the bottom three rows of graphs in Fig. 3. Furthermore, features such as MIC contact time and lymph node activity, shown in Fig. 7 are very comparable between the 1-D and 3-D cases.

**Fig. 8:**
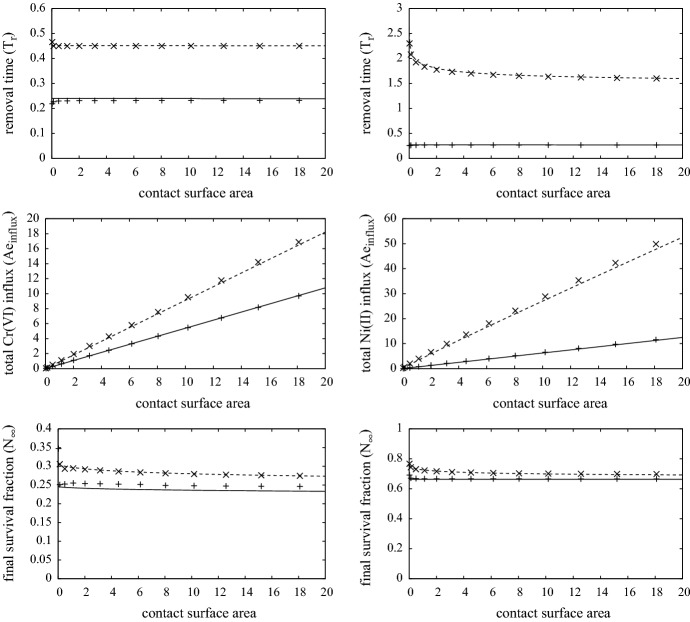
Plots showing the removal time, total ion influx and survival fraction at $t=4$ (steady-state more-or-less reached) against contact surface area following exposure of chromium (left) and nickel (right), with MIC removal occurring when $\min \{N(x,t)\}=0.8$. The dashed and solid curves are solutions of the 1-D model for first and second exposures, respectively, and the “times” and “plus” are the respective solutions of the 3-D model using cylindrical symmetry with contact radius of $0.1, 0.2, 0.4, 0.6, \ldots , 2.4$ units (1 unit being about 1 cm). Parameters are as listed in Table 4

In Sect. 3.3 it is observed in the chromium second contact case that the removal time of MIC is predicted to be fractionally earlier in the 3-D case (about 4%). The reason for this can be seen in the graph of $I_T$ in Fig. 6. Here, there is a notable peak around $r\approx 0.8$, in which the cytokines produced by activated T-cells have drawn immune cells from the periphery to form a chemotactic-driven annulus of inflammation, enhancing locally the skin cell death rate over that in the central contact region. This enhanced cell death leads to earlier MIC removal and in fact leads to an improved outcome in terms of cell survival (about 5%), over the contact region (this is shown in Fig. 8). The differences outlined here are perhaps not too significant for the given parameter set, but the simulation highlights the potential key role of immune cell chemotaxis in inflammatory/allergenic responses and the need for improved in vivo data to determine the relevant parameters.

### Effect of contact surface area

The contact surface area will govern the total mass of the ion that will infiltrate the skin and body, having a particular effect on lymph node activity, as more ions generate greater lymph and T-cell activity according to Eq. (14) and boundary condition (18). The removal time, total ion influx ($Ae_{influx}$) and surviving fraction are shown in Fig. 8 for Cr(VI) (left) and Ni(II)(right) on first (dashed) and second (solid) contact. Here, the overall dimensionless influx is defined to be

29 $$\begin{aligned} Ae_{influx} ~=~ S \int _0^{T_r} w_0 D_{ez} \frac{\partial A_e}{\partial z}(1,t) \, dt, \end{aligned}$$

where *S* is the surface area of contact, noting that $w_0 D_{ey} \partial A_e(1,t) / \partial y$ is the ion influx rate at time *t*.

Increasing the surface area increases the contribution from the integral terms in Eq. (14), so that lymph node activity reaches saturation more rapidly. This effect can be observed in the top and bottom figures where $T_r$ and $N_\infty$ tend to a fixed level beyond 1–2 units squared (equivalent to 1–2 cm$^2$). As noted in the discussion for the 3-D simulation above, the expected close agreement between the 1-D and 3-D simulations follows here. In all cases the $T_r$ and $N_\infty$ are fairly uniform, and only in the Ni(II) first contact case does the graphs notably increase as the contact surface area shrinks. The greatest deviation between the solutions 1-D and 3-D is in the case of second contact with Cr(VI). This is due to the peak in T-cell and innate immune cell distribution near the contact edge due to chemotaxis, that leads to a localisation in skin cell death, and hence the MIC is removed as $N(x,t) = 0.8$ before that which is predicted by the 1-D model. Though the gain from an early removal of MIC is not that significant in the simulations here, it does indicate the possibility that the chemotactic congregation of immune cells which generate excessive localised damage may have a beneficial effect by causing early MIC removal. This will relieve the rest of the skin in contact with the MIC of the worst of the effects of the ion.

**Fig. 9:**
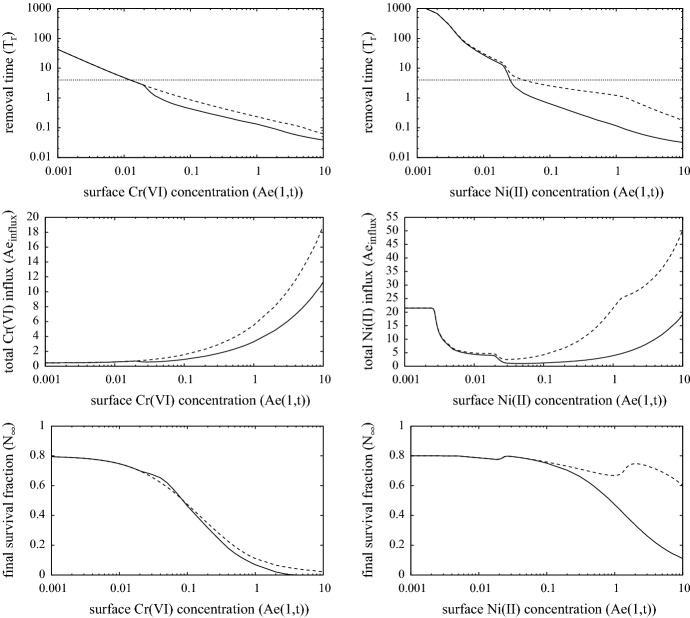
Plots showing the removal time, total $A_e$ infiltration mass and survival fraction as function of external chromium (left) and nickel (right) concentration. The dashed and solid curves are for first and second contact cases. Parameters are as listed in Table 4

### Effect of ion concentration

The rate at which ions can traverse the stratum corneum into the living tissue will depend on, for example, contact location (e.g. corneum thickness), contact material, sweat levels and breaks in skin. So the range of suitable values for the surface ion concentration is likely to be very extensive. Figure 9 shows the effects of surface ion concentration ($A_e(1,t)$) on removal time (when $N=0.8$) and the steady-state levels of overall ion influx and survival fraction ($N_\infty$) following a period of continuous exposure of chromium (left) and nickel (right) on first contact (dashed) and second contact (solid).

We note that the model is only intended to describe events after the first few days of initial contact, so the simulations corresponding to where the removal time $T_r > 4$ (indicated by the dashed line in the top two graphs) are not likely to be biological relevant; it is expected that skin growth will compensate for much of damage in this case, so contact may be tolerated. As expected, the higher concentrations lead to shorter removal time, though the overall influx of ions following contact increases in the case of chromium and to a rather more complicated relationship in the nickel case. In these simulations, T-cells are activated when $A_e > 0.02$, consequently when $A_e(1,t) < 0.02$ then the response is due to the inherent toxicity of the ion and very little immune activity is occurring. In the case of nickel, at surface concentrations in the region of $0.02 \le A_e(1,t) \le 1$, much of the cell death is due to immune cell activity, whilst for $A_e(1,t) > 1$ nickel is sufficiently toxic to displace immune cell activity as the main source of death and consequently “accelerates” removal time; these transitions are evident from the various bumps along the curves for the nickel case. We note that typically more damage occurs on second contact, though there is not a significant difference in the case of chromium.

### Role of skin thickness

**Fig. 10:**
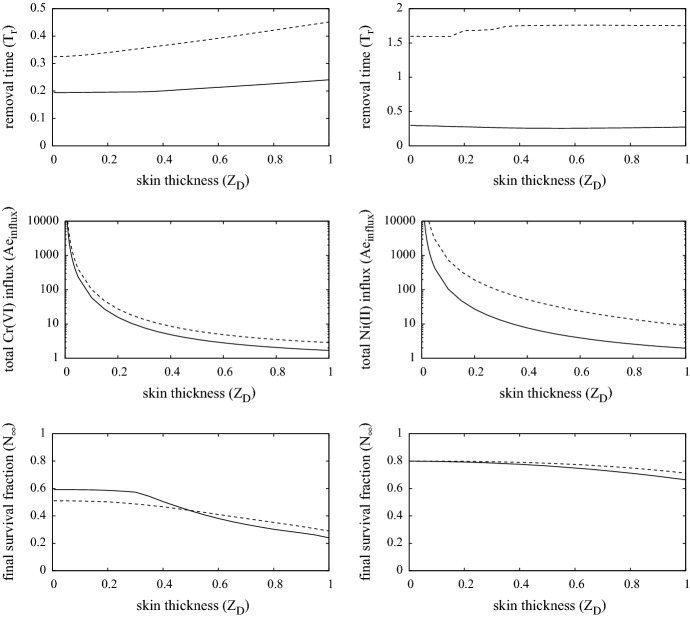
Plots showing the removal time, total $A_e$ infiltration mass and survival fraction as a function of the scaled skin thickness $Z_D$ ($Z_D=1$ is about 2 mm) in response to chromium (left) and nickel (right) ions. The dashed and solid curves are for first and second contact cases. Parameters are as listed in Table 4

The relevant skin thickness, i.e. the distance from the top of the living dermis to the vasculature, will vary considerably depending on the location of MIC contact. The 2 mm assumption ($Z_D=1$) used in the simulations up to now represents the thickest parts of the skin (e.g. on the soles of the feet and palms of the hands). The thinnest would in contrast be about 0.1 mm ($Z_D=0.05$) in the eyelids, for example. Simulated results showing the outcome of MIC contact on skin of varying thicknesses are summarised in Fig. 10. The top row of figures show that the skin thickness does not greatly influence the contact time of the MIC, although it increases slightly as thickness increases. The ion reaches the equilibrium distribution faster in thinner skin, so cells are exposed to higher concentrations faster and hence die slightly faster. This is most notable in the chromium case in which the intrinsic toxicity of the ion is the cause of much of the cell death. The most dramatic change in outcome is observed in the middle row of graphs showing the total influx of the ion. The model is set up so that the ion concentration $A_e$ drops from 0.3 at $z=Z_D$ to zero at $z=0$, consequently the gradient of $A_e$, and hence the flux, increases as the thickness decreases. This, along with the predicted contact time (top row of graphs) being fairly uniform, means that overall $A_e$ influx increases as the thickness decreases; the model thus predicts that the ion infiltrates the body more effectively through thinner skin, which seems reasonable. However, the most interesting prediction from these simulations is that shown in the bottom row of graphs. Here, the surviving fraction is greater where the skin is thinnest. The reason for this observation is that there is a greater capacity in the thicker skin to harbour ions within cells, thus more damage occurs over a longer period; this being most significant in the chromium case. This suggests that thicker skin is the most vulnerable to greater damage in the longer term.

**Fig. 11:**
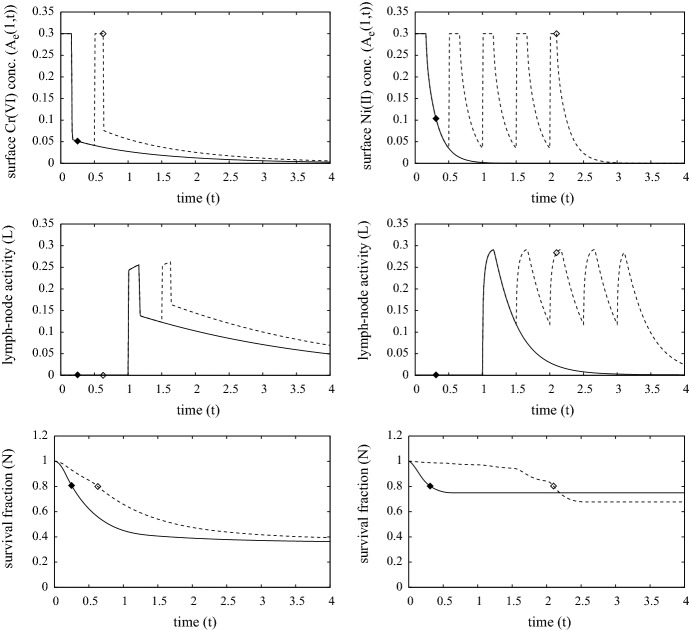
Plots showing the removal time, lymph-node activity and survival fraction against time following repeated exposure of chromium (left) and nickel (right), where dashed and solid curves are first and second exposures, respectively; the time points for permanent MIC removal are indicated by white diamond (first exposure) and black diamond (second exposure). The plots show 8 h exposure ($t=0.167$) followed by 16 h non-exposure until $N=0.8$ when contact ceases. Parameters are as listed in Table 4

### Occupationally relevant exposures

In practice, constant exposure is unlikely unless the metal ions form parts of items such as jewellery or clothing. As discussed in the Introduction, these ions feature in numerous compounds in many workplace situations and typical exposure to these ions will only be for a few hours each day. Figure 11 summarises the main results from a simulation for an exposure cycle of 8 h contact and 16 h non-contact of the MIC until $N = 0.8$ when the MIC was removed permanently. From top to bottom, the graphs show the surface ion concentration $A_e(1,t)$, lymph node activity and cell survival against time for chromium (left) and nickel (right). As expected the point at which $N=0.8$, takes a longer time to reach with the intermittent exposures and thus the long term outcome in terms of cell survival is generally improved. In the second contact case, innate immune and T- cell continued activity after the first removal means that further skin damage occurs to the extent that a second cycle of contact is prevented, consequently, the overall amount of ion introduced into the skin is less than that from continuous contact (compare the solid curves in cell survival fraction graphs in Figs. 3 and 11). A similar circumstance is true for the first contact with chromium, though ion occupation time and general toxicity contributes towards permanent removal before the adaptive response comes into play. For nickel on first contact, its lesser toxicity at the given concentration level means that the exposure can be tolerated for nearly five complete cycles and, interestingly, in this simulation the overall damage exceeds that of the second contact case; we note that this last observation is not universal, but it was observed in a number of simulations during our investigations with parameter values.

**Fig. 12:**
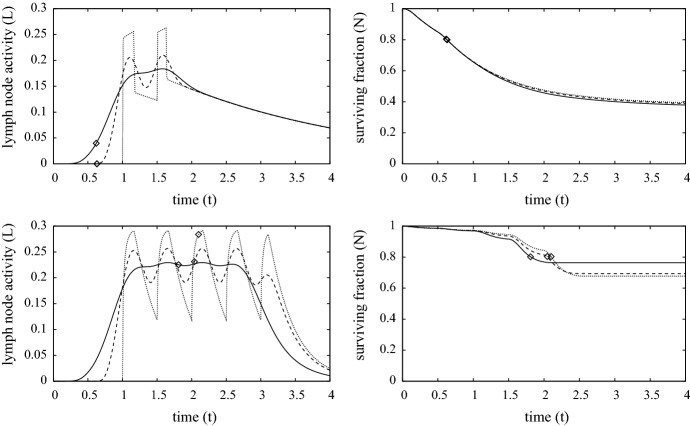
Plots showing the lymph-node activity and survival fraction against time following first-contact exposure of chromium (top) and nickel (bottom), in which a distributive time delay is assumed as defined by Eq. (30). Dotted curves are for $\sigma = 0$ (i.e. that used in all other simulations), the dashed and solid curves are for $\sigma = 0.1$ ($\approx 2.4$ h) and $\sigma = 0.2$ ($\approx 4.8$ h), respectively. The time point at which the MIC is removed are indicated by a white diamond. Parameters are as listed in Table 4

### Effect of distributional time delay

The sharp jumps in lymph node activity seen in previous figures is due to the relatively rapid equilibration of external ion distribution on application and removal of the MIC, coupled with the single point time delay for the adaptive process used as first approximation. In reality, such jumps are unlikely to be so dramatic due to time variations in activities of dendritic cell (i.e. activation, migration to and occupancy as antigen presenters in lymph nodes) and programmed T-cells (i.e. proliferation in and release from lymph nodes). This motivated the inclusion in our model of a distributional time delay terms in Eq. (14) as a simple way to account for these time variations. Simulations investigating the effect of a variable response time with a mean of 2 days ($t=1$) are shown in Fig. 12 for first contact of the metal ion in the “occupationally relevant” exposure case. Here, the timescale for the entire adaptive immune response process is assumed to satisfy a truncated normal distribution, thereby the kernel $\kappa (\tau )$ in Eq. (14) is defined as

30 $$\begin{aligned} \kappa (\tau ) ~=~ \frac{e^{-(\tau -1)^2/\sigma ^2}}{\int _{1-\omega }^{1+\omega } \exp (-(\tau -1)^2/\sigma ^2) \, d \tau }, \end{aligned}$$

for $\tau \in (1-\omega , 1+\omega )$, where $\sigma$ is the standard deviation of response time about the mean $\tau =1$ and $\omega < 1$ is an assumed maximum variation (in these simulations, $\omega$ was chosen so that $e^{-\omega ^2/\sigma ^2} = 10^{-8}$). The distributive time delay has a marked consequence on predicted lymph node activity, where the sharp jumps of the dotted curves are smoothed out significantly as the variation $\sigma$ increases. This has little effect in the chromium case as the toxicity of the ion leads to the removal of the MIC and consequently there is little difference in cell survival predicted. However, in the case of nickel, the greater variation ($\sigma =0.2$) leads to an earlier removal of the source of metal ion (about a day, solid line in bottom left figure) in comparison to that of the lesser variation cases. The main reason for this is that a non-negligible amount of T-cells are being released by the lymph node earlier and able to be sufficiently active in the contact zone to cause the early response. The consequence of the early removal can be seen in the bottom right figure in which the cell survival is improved by 10–15% from the early removal.

## Summary and discussion

The influx of metal ions through skin contact with metal, or products containing metals, leads to a complex response both directly, via toxic activity on cells, and indirectly, via immunological activity. Furthermore, different ionic species, Cr(VI) and Ni(II) being relevant to this paper, can produce significantly different skin reactions as can the history of contact from the same species. Much of what we know regarding cellular response to these ions is from in vitro studies, but how these responses interact in situ to result in dermatitis is much less well understood. The aim of this paper was to formulate a mathematical model which incorporates many of the important known factors that lead to dermatitis development in a spatio-temporal setting, that can offer insights as to why there are differences in skin responses between two ionic species, namely Cr(VI) and Ni(II), and between first and second contact. In the interest of keeping the model manageable, we reduced the complexity by focusing on metal ion toxicity (using data from Franks et al. 2008), the innate and adaptive immune response and regulation via a single, generic cytokine. Despite the number of simplifications in the modelling assumptions, the model possesses a large number of parameters. In the simulations we used values that are available from the literature. Uninformed parameters were estimated and were tuned to produce results showing the desired differences in skin response to differing circumstances.

The simulations discussed in Sects. 3.1 and 3.2 shows many of the expected features of the exposure to each metal and makes some predictions that would enable comparison with data. At the ion levels used in the simulations, Cr(VI) is more toxic than Ni(II). On first contact, this toxicity, together with the innate response, leads to fairly rapid skin damage and subsequent removal of the Cr(VI) yielding irritant prior to an adaptive response. For Ni(II), the lack of toxicity and cytokine production by the affected skin cells, means that the innate immune response occurs at a very low level until a sufficient amount of T-cell infiltrate the affected area. On second contact, however, the toxicity of ion is fairly secondary as the T-cell response and the consequent cytokine mediated activation of the immune cells dominate leading to a faster removal of irritant. There are a number of predictions that could be investigated further clinically based on the results presented here,

1. Cr(VI) seems to linger in the skin longer than that of Ni(II), leading to prolonged damage and lymph-node/T-cell mediated activity.
2. To generate the large response difference between first and second contact cases for Ni(II), the T-cell infiltration rate has to be relatively slow (governed by parameter $Q_T$) and T-cells have to die off slowly (i.e. small death rate $\delta _T$); thus ion-specific T-cells are predicted to occupy the contact site for a long time (i.e. weeks) after the contact has been removed.
3. The typically small aspect ratio of skin depth to contact surface area means that there is little difference in the overall outcomes predicted by the 1-D and the radially symmetric 3-D simulations. However, latter simulations do hint at the possibility of extra skin damage occurring in a local region near the vicinity of the contact edge; this being due to the chemotactic infiltration of immune cells from the contact region periphery.

An extensive parameter exploration was undertaken in order to obtain the “standard values” listed Table 4, which results with model solutions that are consistent with observation and expectation. Our investigations found that small deviations from these values ($< 25\%$) do not change the qualitative, or significantly the quantitative, behaviour of the results. The focus of the simulations presented in Sects. 3.3–3.6 were on the parameters that govern factors most testable experimentally, such as contact area, ion surface concentration (reflecting different means of ion delivery), contact location (reflected by skin depth) and intermittent “occupationally relevant” contacts. In Sect. 3.7 we investigated whether or not smoothing out the rather unlikely “abrupt switching” of lymph node activity would have any significant effect on the results. There are a number of interesting observations from the results of these simulations and we list the salient points.

1. The contact surface area has little effect on removal time. This is partly due to our criteria for metal ion carrier (MIC) removal being when there is 20% localised damage and that over the contact surface distributions of ions and immune cells are fairly uniform (see 3-D simulations). However, the larger surface area means that more ions enter the body, which may lead to other detrimental effects.
2. Removal time only decreases by a small amount as skin thickness decreases and that thicker skin will experience greater damage in the longer term.
3. Intermittent contact appears not to make a significant difference in skin response qualitatively, though, due to the lingering of ions in the skin, the overall contact time of irritant will be less than it would be for continuous contact.
4. Assuming a single point or a simple distributed time delay in lymph-node activity does not effect results too much, particularly in the continuous contact case (results not shown). However, with intermittent contact case, it is possible that a spreading of response can lead to earlier removal.

Further to this, there are two key questions highlighted from the investigation in Sect. 3.4, namely (1) What is the ion concentration in the skin? and (2) At what ion concentration do targeted T-cells “activate” and generate an immune response. In the simulations a surface concentration of 0.3 $\upmu$M and activation level of $A_{e_i} = 0.02~\upmu$M was assumed, which ensures that immune activity will cause skin damage even at nontoxic ion levels; how this reflects reality is unknown.

The parameter gaps in the current model are mainly centred around the various immune cells, such as diffusion and chemotaxis rate coefficients in tissues, typical concentrations of cytotoxic granules and production rates and cytokine or ion concentration levels for immune cell activation. Estimates of these parameters from, if possible, in vitro studies would be invaluable for model verification and improvement, which will help assess the adequacy of the current assumptions and/or the need to include other factors to formulate a more dependable model. In our interest of making a detailed model as simple as possible, we made a number of simplifications to reality. These include the blending of epidermis and upper dermis into homogeneous living tissue, absence of skin regrowth and recovery, assuming a single pro-inflammetory cytokine etc.; including these mechanisms will doubtless improve the model, but will come at a cost of more, as yet undetermined, parameters. Nevertheless, the current model has made a number of interesting predictions, which will hopefully motivate further and more directed investigations into contact dermatitis.

## A Non-dimensionalisation

We focus on the radial symmetric 3-dimensional case and non-dimensionalise the model using the scalings

$$\begin{aligned}&t={\bar{\tau }} \,{\hat{t}}, \qquad r=R\,{\hat{r}}, \qquad z=Z\,{\hat{z}}, \\&A_e = A_0 \,{\hat{A}}_e, ~ A_n=A_0\, {\hat{A}}_i,~A_p=A_0 {\hat{A}}_p, ~ I=I^*\,{\hat{I}},\\&\quad T=T^*\, {\hat{T}},~c= c_{ss} \,{\hat{c}}, ~ g=g^* \,{\hat{g}}, \end{aligned}$$

where

$$\begin{aligned} I^*= & {} \frac{Q_I(1-w_0)}{\sqrt{\delta _{{\mathrm{I}}} D_I}} \coth \left( \sqrt{\frac{\delta _{{\mathrm{I}}}}{D_I(1-w_0)}} \, Z\right) \\ T^*= & {} \varepsilon \,\frac{ Q_T(1-w_0)}{\sqrt{\delta _{{\mathrm{T}}} D_T}} \coth \left( \sqrt{\frac{\delta _{{\mathrm{T}}}}{D_T(1-w_0)}} \, Z\right) , \end{aligned}$$

represents a suitable normalisation of *I* and *T* at $t=0$ for the $L_0=\epsilon$ case; note that from the data discussed in Table 2 and 3, $I_*=I_H \approx 10^{-10} \mu$M and $T_*=T_H \approx 10^{-16} \mu$M. Time has been scaled with the delay term in Eq. (14) whereby ${\hat{t}}=1$ is equivalent to approximately 2 days. A suitable choice of scaling for *g* is not entirely clear, but we opted to choose $g^*=\beta _{{\mathrm{gc}}} c_{ss} I^* {\bar{\tau }}$ so that the dimensionless equivalent of $\beta _{{\mathrm{gc}}}$ is 1.

The scalings for *c*, *I* and *T* leads to

31 $$\begin{aligned} {\hat{\delta }}_{\mathrm{c}}= & {} {\hat{\beta }}_{\mathrm{cn}}=\frac{{\bar{\tau }}}{w_0c_{ss}} \beta _{{\mathrm{cn}}}=\frac{{\bar{\tau }} \delta _{{\mathrm{c}}}}{1\!-\!w_0},~~~~ {\hat{\beta }}_{\mathrm{gc}}=\frac{I_* {\bar{\tau }}}{g^*} \beta _{{\mathrm{gc}}} ~=~ 1,\nonumber \\ {\hat{Q}}_I= & {} \frac{{\bar{\tau }}}{I^* Z} Q_I = \frac{{\bar{\tau }}}{Z} \sqrt{\frac{{\hat{\delta }}_{\mathrm{I}}{\hat{D}}_{Iz}}{1\!-\!w_0}} \tanh \!\left( \sqrt{\frac{{\hat{\delta }}_{\mathrm{I}}}{{\hat{D}}_{Iz}(1\!-\!w_0)}}\right) , \nonumber \\ {\hat{Q}}_T= & {} \frac{{\bar{\tau }}}{T^* Z } Q_T = \frac{{\bar{\tau }}}{\varepsilon Z} \sqrt{\frac{{\hat{\delta }}_{\mathrm{T}}{\hat{D}}_{Tz}}{1\!-\!w_0}} \tanh \!\left( \sqrt{\frac{{\hat{\delta }}_{\mathrm{T}}}{{\hat{D}}_{Tz}(1\!-\!w_0)}}\right) , \end{aligned}$$

and the remaining dimensionless parameters are defined as

$$\begin{aligned}&{\hat{\beta }}_{\mathrm{ci}}=\frac{A_0 {\bar{\tau }}}{w_0 c_{ss}}\beta _{{\mathrm{ci}}},~ {\hat{\beta }}_{\mathrm{I}} = {\bar{\tau }} \beta _{{\mathrm{I}}}, ~ {\hat{\beta }}_{\mathrm{T}}={\bar{\tau }} \beta _{{\mathrm{T}}}, ~ {\hat{\beta }}_{\mathrm{cET}} = \frac{{\bar{\tau }} T^* \beta _{{\mathrm{cET}}}}{c_{ss}},~ {\hat{\beta }}_{\mathrm{cI}}=\frac{I^* {\bar{\tau }}}{c_{ss}} \beta _{{\mathrm{cI}}}, \\&{\hat{\beta }}_{\mathrm{gi}}=\frac{A_0 I_* {\bar{\tau }}}{g^*} \beta _{{\mathrm{gi}}},~ {\hat{c}}_T=\frac{c_T}{c_{ss}},~ {\hat{c}}_I=\frac{c_I}{c_{ss}},~ {\hat{M}}_c=\frac{I_*}{g^*}M_c,~ {\hat{M}}_g=g^*M_g, \\&{\hat{\delta }}_{\mathrm{ni}}=A_0 {\bar{\tau }}\delta _{{\mathrm{ni}}},~ {\hat{\delta }}_{\mathrm{nTi}}=T^* A_0 {\bar{\tau }} \delta _{{\mathrm{nTi}}},~ {\hat{\delta }}_{\mathrm{ng}}= g^* {\bar{\tau }} \delta _{{\mathrm{ng}}},~ {\hat{\delta }}_{\mathrm{eD}}={\bar{\tau }} \delta _{{\mathrm{eD}}}, \\&{\hat{\delta }}_{\mathrm{c}}={\bar{\tau }} \delta _{{\mathrm{c}}},~ {\hat{\delta }}_{\mathrm{I}}={\bar{\tau }} \delta _{{\mathrm{I}}},~ {\hat{\delta }}_{\mathrm{g}}={\bar{\tau }} \delta _{{\mathrm{g}}}, ~ {\hat{\delta }}_{\mathrm{T}}={\bar{\tau }} \delta _{{\mathrm{T}}}, \\&{\hat{A}}_{surface} = \frac{A_{surface}}{A_0},~ \hat{k_n}={\bar{\tau }} k_n,~ {\hat{k}}_p={\bar{\tau }} k_p,\\&\quad {\hat{K}}_{L_i}=\frac{K_{L_i}}{A_0 w_0 V_0},~ {\hat{\lambda }}=\lambda {\bar{\tau }}, {\hat{Q}}_c = \frac{Q_c {\bar{\tau }}}{Z},\\&{\hat{D}}_{ir}=\frac{{\bar{\tau }}}{R^2} D_i,~ {\hat{D}}_{iz}=\frac{{\bar{\tau }}}{Z^2} D_i,~ {\hat{D}}_{jr}=\frac{{\bar{\tau }}}{R^2} D_{j},~ {\hat{D}}_{jz}=\frac{{\bar{\tau }}}{Z^2} D_{j},\\&{\hat{\chi }}_{i r} = \frac{{\bar{\tau }} c_{ss}}{R^2}\chi _{i r}, ~ {\hat{\chi }}_{i z} = \frac{{\bar{\tau }} c_{ss}}{Z^2}\chi _{i z}, \end{aligned}$$

where $i=I,T$ and $j=e,c$ and the kernel function becomes $\kappa (\tau )=\kappa ({\bar{\tau }} {\hat{\tau }}) = {\hat{\kappa }}({\hat{\tau }})/{\bar{\tau }}$ (here, the denominator will divide out from the rescaling of the time variable of the integral). These rescalings, on dropping the hats, lead to the following dimensionless system of equations

32 $$\begin{aligned} \frac{\partial n}{\partial t}= & {} -K_d\,n, \end{aligned}$$

33 $$\begin{aligned} p= & {} 1-w_0- n . \end{aligned}$$

34 $$\begin{aligned} \frac{\partial (nA_n)}{\partial t}= & {} -k_nn(A_n - \mu _n A_e) \,-\, K_d nA_n, \end{aligned}$$

35 $$\begin{aligned} \frac{\partial (pA_p)}{\partial t}= & {} -k_p p(A_p - \mu _p A_e) \,+\, K_d nA_n, \nonumber \\ w_0 \frac{\partial A_e}{\partial t}= & {} w_0 \nabla \cdot \left( \underline{\underline{D_e}} \nabla A_e \right) \,+ \,k_n n(A_n - \mu _n A_e) + k_p p(A_p - \mu _p A_e) -\delta _{{\mathrm{eD}}} A_e, \nonumber \\\end{aligned}$$

36 $$\begin{aligned} \frac{\partial c}{\partial t}= & {} \nabla \cdot \left( \underline{\underline{D_{c}}} \nabla c \right) \,+\, \beta _{{\mathrm{ci}}} n A_n \,+ \,\beta _{{\mathrm{cET}}} \frac{A_e}{(A_{c_i}+A_e)} T \,+ \, \beta _{{\mathrm{cI}}} H(c-c_I) I \nonumber \\&\,+\, \delta _{{\mathrm{c}}}\left( \frac{n}{1\!-\!w_0}-c\right) \!, \end{aligned}$$

37 $$\begin{aligned} \frac{\partial I}{\partial t}= & {} \nabla \cdot \left( n \underline{\underline{D_{I}}} \nabla I \,-\, n\underline{\underline{\chi _{I}}} H(c-c_{I})I\nabla c\right) \, + \,\beta _{I} H(c-c_I) I \nonumber \\&-~\left( \frac{(H(c-c_I)+\beta _{{\mathrm{gi}}} nA_n)}{M_c} \,+\, \delta _{{\mathrm{I}}}(1-\delta _{Ic}H(c-c_I))\right) I, \end{aligned}$$

38 $$\begin{aligned} \frac{\partial g}{\partial t}= & {} (H(c-c_I)+\beta _{{\mathrm{gi}}} nA_n)I \,-\,\frac{\delta _{{\mathrm{ng}}}}{M_g} gn-\delta _{{\mathrm{g}}} g, \end{aligned}$$

39 $$\begin{aligned} \frac{\partial T}{\partial t}= & {} \nabla \cdot \left( n \underline{\underline{D_{T}}} \nabla T \,-\, n\underline{\underline{\chi _{Ti}}} H(F(A_e,c)-1)T\nabla c\right) \nonumber \\&+ (\beta _{{\mathrm{T}}} H(F(A_e,c)\!-\!1) - \delta _{{\mathrm{T}}}) T, \end{aligned}$$

40 $$\begin{aligned} \frac{dL}{dt}= & {} \lambda \left( \frac{\displaystyle {\int _V \int _0^\infty \kappa (\tau ) A_e(\varvec{x},t-\tau ) \,\text{ d }\tau \,\text{ d }V}}{\displaystyle {K_{L_i}+\int _V \int _0^\infty \kappa (\tau ) A_e(\varvec{x},t-\tau ) \,\text{ d }\,\tau \text{ d }V}} \,+\, L(\varepsilon -L) \right) , \end{aligned}$$

where the tensors $\underline{\underline{D_j}}$ and $\underline{\underline{\chi _k}}$ ($j=A_e,c,I,T$ and $k=I,T$) are diagonal matrices with elements $D_{j*}$ and $\chi _{k*}$ multiplying the derivative with respect to variable $*$, and

41 $$\begin{aligned} K_d ~=~ (\delta _{{\mathrm{ni}}}+\delta _{{\mathrm{nTi}}}H(F(A_e,c)\!-\!1)T)A_n \,+\, \delta _{{\mathrm{ng}}}g. \end{aligned}$$

These are subject to

42 $$\begin{aligned} \text{ at } t=0&n=1-w_0,~ I=I_I(z),~ T= T_I(z;L_0),~L=L_0,~ A_n=0, \nonumber \\&A_e=0,~p=0,~p_g=0,~c=c_I(z),~g=0, ~A_p=0, \end{aligned}$$

43 $$\begin{aligned} \text{ at } z=0&A_e=0,~D_{cz} \frac{\partial c}{\partial z}=Q_c c, ~-D_{Iz} \frac{\partial I}{\partial z}= Q_I, ~ -D_{Tz} \frac{\partial T}{\partial z}= Q_T\,L, \end{aligned}$$

44 $$\begin{aligned} \text{ at } z=1&\frac{\partial c}{\partial z} = \frac{\partial I}{\partial z}=\frac{\partial T}{\partial z}=0,~ \left\{ \begin{array}{ll} A_e=A_{surface} ~~&{} 0 \le x \le 1\\ \frac{\partial A_e}{\partial z}=0 &{} x> 1 \end{array} \right. \end{aligned}$$

45 $$\begin{aligned} \text{ at } r=0&\frac{\partial A_e}{\partial r}=\frac{\partial c}{\partial r} = \frac{\partial I}{\partial r}=\frac{\partial T}{\partial r}=0, \end{aligned}$$

46 $$\begin{aligned} \text{ as } r \rightarrow \infty&A_e\rightarrow 0,~c\rightarrow c_I(z), ~I\rightarrow I_I(z),~T\rightarrow T_\infty (z,t), \end{aligned}$$

where

47 $$\begin{aligned} I_{I}(z)= & {} \cosh \left( \gamma _{I} (1-z)\right) / \cosh \left( \gamma _{I}\right) , \end{aligned}$$

48 $$\begin{aligned} T_{I}(z; L_0)= & {} L_{0} \,\cosh \left( \gamma _{T}(1-z)\right) / \cosh \left( \gamma _{T}\right) , \end{aligned}$$

49 $$\begin{aligned} c_{I}(z)= & {} 1 - \frac{Q_{c} \cosh \left( \gamma _{c} (1-z)\right) }{Q_{c} \cosh \left( \gamma _{c} \right) + \sqrt{D_{c} \delta _{c}}\sinh \left( \gamma _{c}\right) }, \end{aligned}$$

where $L_0$ is defined by (26) and $\gamma _k = (\delta _k/D_{kz}(1-w_0))^{1/2}$ for $k=I, T$ and $\gamma _c = (\delta _c/D_{cz})^{1/2}$. Here $T_\infty$ is defined by

50 $$\begin{aligned} \frac{\partial T_\infty }{\partial t}= & {} D_{Tz}(1-w_0) \frac{\partial ^{2} T_\infty }{\partial z^2} \,-\,\delta _{{\mathrm{T}}} T_\infty , \end{aligned}$$

subject to $-D_{Tz}\partial T_\infty / \partial z=Q_T L$ at $z=0$ and $\partial T_\infty / \partial z=0$ at $z=1$.
